# Effect of age on the sensitivity of the rat thyroid gland to ionizing radiation

**DOI:** 10.1093/jrr/rrv003

**Published:** 2015-02-16

**Authors:** Mutsumi Matsuu-Matsuyama, Kazuko Shichijo, Kumio Okaichi, Tomomi Kurashige, Hisayoshi Kondo, Shiro Miura, Masahiro Nakashima

**Affiliations:** 1 Tissue and Histopathology Section, Atomic Bomb Disease Institute, Nagasaki University, 1-12-4 Sakamoto, Nagasaki 852-8523, Japan; 2 Department of Tumor and Diagnostic Pathology, Atomic Bomb Disease Institute, Nagasaki University, 1-12-4 Sakamoto, Nagasaki 852-8523, Japan; 3 Department of Radioisotope Medicine, Atomic Bomb Disease Institute, Nagasaki University, 1-12-4 Sakamoto, Nagasaki 852-8523, Japan; 4 Department of Molecular Medicine, Atomic Bomb Disease Institute, Nagasaki University, 1-12-4 Sakamoto, Nagasaki 852-8523, Japan; 5 Biostatistics Section, Atomic Bomb Disease Institute, Nagasaki University, 1-12-4 Sakamoto, Nagasaki 852-8523, Japan

**Keywords:** rat, thyroid, X-ray, p53 binding protein 1, apoptosis, autophagy

## Abstract

Exposure to ionizing radiation during childhood is a well-known risk factor for thyroid cancer. Our study evaluated the effect of age on the radiosensitivity of rat thyroid glands. Four-week-old (4W), 7 -week-old (7W), and 8-month-old (8M) male Wistar rats were exposed to 8 Gy of whole-body X-ray irradiation. Thyroids were removed 3–72 h after irradiation, and non-irradiated thyroids served as controls. Ki67-positivity and p53 binding protein 1 (53BP1) focus formation (a DNA damage response) were evaluated via immunohistochemistry. Amounts of proteins involved in DNA damage response (p53, p53 phosphorylated at serine 15, p21), apoptosis (cleaved caspase-3), and autophagy (LC3, p62) were determined via western blotting. mRNA levels of 84 key autophagy-related genes were quantified using polymerase chain reaction arrays. Ki67-positive cells in 4W (with high proliferative activity) and 7W thyroids significantly decreased in number post-irradiation. The number of 53BP1 foci and amount of p53 phosphorylated at serine 15 increased 3 h after irradiation, regardless of age. No increase in apoptosis or in the levels of p53, p21 or cleaved caspase-3 was detected for any ages. Levels of LC3-II and p62 increased in irradiated 4W but not 8M thyroids, whereas expression of several autophagy-related genes was higher in 4W than 8M irradiated thyroids. Irradiation increased the expression of genes encoding pro-apoptotic proteins in both 4W and 8M thyroids. In summary, no apoptosis or p53 accumulation was noted, despite the expression of some pro-apoptotic genes in immature and adult thyroids. Irradiation induced autophagy in immature, but not in adult, rat thyroids.

## INTRODUCTION

Exposure to ionizing radiation (IR), especially during childhood, is a well-known risk factor for thyroid cancer. Recent studies of atomic bomb survivors suggest that persons exposed early in life have a very high risk of thyroid cancer, but the risk decreases with attained age [1]. Richardson found that exposure to IR in adulthood was positively associated with thyroid cancer among female but not male atomic bomb survivors [2]. Changes in the radiation sensitivity of the thyroid epithelium between childhood and adulthood have not been clearly determined.

Ataxia telangiectasia mutated (ATM) is the primary detector of DNA damage due to DNA double-strand breaks (DSBs) after exposure to IR. When activated via phosphorylation upon DNA damage, ATM phosphorylates the key proteins involved in the DNA damage response such as 53BP1 (p53-binding protein 1) and p53 [3]. 53BP1 molecules localize at DSBs and rapidly form nuclear foci [4]. 53BP1 focus formation is considered a cytologic marker of DSBs [5]. Exposure to IR results in rapid apoptosis in actively proliferating tissues such as bone marrow, spleen, thymus, and small intestine [6, 7]. The most extensively studied tumor suppressor is p53, which promotes cell-cycle arrest, DNA repair, cellular senescence, and apoptosis in response to diverse forms of cellular stress including DNA damage [8]. When active, ATM phosphorylates p53 at serine 15 (phospho-p53^Ser15^), which is critical for p53 activation in response to IR-induced DNA damage [9, 10]. p53 accumulates and translocates to the nucleus, where it binds to DNA to transactivate numerous genes, including those encoding p21^WAF1/CIP1^(p21), Bax, Noxa and Puma. p21 induces G1 arrest by inhibiting the activity of cyclin-dependent kinases [11, 12]. Bax, Noxa and Puma are apoptotic proteins that localize to mitochondria to promote the release of cytochrome C from mitochondria, which is required for the cleavage and consequent activation of caspases [13]. Caspase 3, one of the primary executioners of apoptosis, is necessary for the cleavage of a large number of proteins, margination of apoptosis-associated chromatin, DNA fragmentation, and nuclear collapse during apoptosis [14]. Recently, it was reported that 8 Gy of X-ray irradiation induced apoptosis in the crypt cells of the rat small intestine, accompanied by accumulation of p53, induction of p21 and Puma, and caspase 3 cleavage [15]. Basic fibroblast growth factor protected cells from radiation-induced apoptosis via suppression of the p53 pathway [15]. Other studies, however, suggest that external exposure to 8 Gy of IR does not lead to apoptosis of thyroid epithelial cells *in vivo* [16]. The adult thyroid cell population turns over slowly, with cell loss compensating for proliferation [17].

Autophagy is a non-apoptotic form of cell elimination [18]. It is a highly regulated process involving bulk lysosome-mediated degradation of cytoplasmic macromolecules and organelles in cells during starvation, differentiation, and normal growth to maintain cellular homeostasis and survival [19–21]. LC3 is a marker of autophagy, and its conversion from LC-I to LC-II is required for autophagosome formation [22]. The p62 protein acts as a molecular adaptor between the autophagic machinery and its substrates [23]. It is unclear whether autophagy is an early response to irradiation in thyroid follicular epithelial cells.

To evaluate the effect of age on the radiosensitivity of rat thyroid follicular epithelial cells after irradiation *in vivo*, cell proliferation, expression of DNA damage response molecules (including those in the p53 pathway), and induction of apoptosis were determined in 4-week-old (4W), 7-week-old (7W) and 8-month-old (8M) rat thyroids after 8 Gy whole-body X-ray irradiation. We used male rats in this study to investigate the effects of radiation (without the influence of other factors such as female hormones) on thyroid tissues. The abundance of LC3-II and p62 was quantified to determine whether autophagy was induced in rat thyroids after irradiation. Furthermore, to determine whether radiation induced the expression of autophagy genes, we analyzed the mRNA levels of a group of 84 key autophagy-related genes using polymerase chain reaction (PCR) arrays [24].

## MATERIALS AND METHODS

### Animals

4W immature (90–110 g, *n* = 62), 7W young adult (240–280 g, *n* = 44), and 8M adult (570–710 g, *n* = 36) male Wistar rats were purchased from Charles River Japan (Atsugi, Japan). All animals were kept in a pathogen-free facility at the Nagasaki University Center for Frontier Life Sciences in accordance with the rules and regulations of the Institutional Animal Care and Use Committee.

### Irradiation

Irradiation was performed between 9:00 a.m. and noon. 4W (*n* = 46), 7W (*n* = 33), and 8M (*n* = 30) rats received 8 Gy of whole-body X-ray irradiation using a Toshiba ISOVOLT TITAN32 X-ray, 200 kV, 15 mA apparatus with 0.5-mm aluminum + 0.5-mm copper + 5-mm aluminum filters at a dose rate of 0.5531 Gy/min. One or two rats were treated simultaneously while being held in a cardboard box. Control rats were non-irradiated but were otherwise handled identically (4W, *n* = 16; 7W, *n* = 11; 8M, *n* = 6).

### Paraffin-embedded tissue preparation

Thyroid tissues were removed at 3, 6, 24, 48 and 72 h after irradiation, after sacrificing rats via deep anesthesia. Non-irradiated thyroid tissues and the thymus (radiosensitive control) were also removed. Tissue samples were fixed overnight in a 10% formalin solution. Thyroid and thymus tissues were embedded in paraffin blocks, and 3-µm sections were cut and slide sections prepared.

### Ki67 immunohistochemistry

Deparaffinized sections were pretreated for antigen retrieval via scientific microwave treatment in 0.01 mol/l citrate buffer (pH 6.0). After a 10-min incubation in 3% H_2_O_2_ in deionized water to inhibit endogenous peroxidase activity, the sections were incubated with anti-rat Ki67 monoclonal antibody (MIB-5) diluted 1:50 in ChemMate antibody diluent. After washing with phosphate-buffered saline (PBS), the sections were incubated with biotinylated anti-rabbit and anti-mouse immunoglobulins for 30 min and subsequently with streptavidin-conjugated horseradish peroxidase for 30 min using an LSAB-2 system-HRP. Antibody binding was visualized via incubation of the sections with 3,3′-diaminobenzidine (DAB) using a liquid DAB^+^ substrate chromogen system. The percentage of Ki67-positive cells was determined in a minimum of five fields per rat for three to eight rats for each datapoint using light microscopy ( × 400 magnification). Ki67 antibody and ChemMate antibody diluent were obtained from DAKO, Denmark A/S (Glostrup, Denmark), and the LSAB-2 system-HRP and liquid DAB^+^ substrate chromogen system were obtained from DAKO North America, Inc. (Carpinteria, CA).

### 53BP1 immunofluorescence staining

After microwave treatment in citrate buffer, deparaffinized sections were preincubated with blocking buffer (1% bovine serum albumin in PBS) for 30 min. Sections were incubated overnight with anti-53BP1 polyclonal antibody (Bethyl Labs, Montgomery, TX) at a 1:200 dilution at 4°C followed by incubation with Alexa Fluor 488-conjugated goat anti-rabbit antibody (Invitrogen, Carlsbad, CA). Sections were counterstained with 4′,6-diamidino-2-phenylindole dihydrochloride (Vector Laboratories, Burlingame, CA), and visualized and photographed using a fluorescence microscope (BZ-9000, KEYENCE, Osaka, Japan). 53BP1 foci were counted in six fields per rat for three to seven rats for each datapoint ( × 1000 magnification).

### Terminal deoxynucleotidyl transferase-mediated dUTP nick end labeling staining

Terminal deoxynucleotidyl transferase-mediated dUTP nick end labeling (TUNEL) staining was performed using the ApopTag Peroxidase In Situ Apoptosis Detection kit (Millipore, Temecula, CA). Sections were deparaffinized and digested with proteinase K (36 µg/ml) for 15 min at room temperature. Endogenous peroxidase activity was blocked with 3% hydrogen peroxide. Sections were incubated with terminal deoxynucleotidyl transferase in reaction buffer containing a fixed concentration of digoxigenin-labeled nucleotides for 1 h at 37°C; reactions were terminated by incubating slides in Stop/Wash buffer for 10 min. The sections were incubated with anti-digoxigenin peroxidase for 30 min, and apoptotic cells were detected after incubation in DAB for ∼10 min and counterstaining with methyl green (Sigma–Aldrich, St Louis, MO). The percentage of TUNEL-positive cells was determined in a minimum of five fields per rat from three to seven rats for each datapoint ( × 400 magnification).

### Western blotting

Thyroid and thymus samples at 3, 6 and 24 h after 8 Gy irradiation were used for detection of p53, phospho-p53^Ser15^, p21, and cleaved caspase-3. Thyroid samples at 3, 6, 24, 48 and 72 h after irradiation were used for detection of LC3 and p62. Non-irradiated thyroid and thymus samples (controls) were frozen immediately after removal. Total protein was extracted from tissue samples as described previously [25]. Proteins (30 µg) were resolved on 10% or 15% SDS-polyacrylamide gels and transferred electrophoretically to Hybond ECL nitrocellulose membranes (Amersham, Arlington Heights, IL). Membranes were incubated with antibodies to p53 (Pab421) (Oncogene Science Inc., Uniondale, NY), p21 (Santa Cruz Biotechnology, Santa Cruz, CA), phospho-p53^Ser15^, cleaved caspase 3 (Cell Signaling Technology Inc.), LC3, p62/SQSTM1 (MBL, Nagoya, Japan), or actin (Sigma–Aldrich, St Louis, MO). Proteins recognized by the antibodies were visualized via chemiluminescence (ECL Plus, Amersham), and the densities of the protein bands were quantified using a luminescent image analyzer (LAS4000) (Fujifilm, Tokyo, Japan) and NIH Image J software. The amount of protein was normalized to the amount of actin. Data are expressed as fold change in protein level relative to the level in the non-irradiated control (0 h). LC3 levels are expressed as fold change in the LC3-II/LC3-I ratio relative to the ratio in the non-irradiated control.

### RNA extraction, reverse transcription, and quantitative PCR

Thyroid samples of 4W and 8M rats 24 h after 8 Gy irradiation and non-irradiated thyroid samples were incubated in RNA Stabilization Reagent for 24 h at 4°C and stored at −80°C. Total RNA was extracted using QIAzol Lysis Reagent and the RNeasy Mini Kit according to the manufacturer's instructions. cDNA synthesis and reverse transcription were performed using the RT^2^ First Strand Kit and 1 μg of total RNA. cDNA (102 µl) was mixed with 1.35 ml of 2 × RT^2^ SYBR Green Master Mix and RNase-free water to a final volume of 2.7 ml. Each well in the RT^2^ Profiler PCR Array plate contained a 25-µl sample; samples were analyzed in triplicate. The PCR array contained 84 primer pairs that amplify genes involved in rat autophagy. Reactions were performed using a Takara TP-800 thermal cycler (Takara, Shiga, Japan) following the manufacturer's instructions. All kits and reagents and the PCR array (catalog no. PARN-084ZA) were purchased from Qiagen (Tokyo, Japan). Fold changes in expression were calculated using the ΔΔCt method (http://www.SABiosciences.com/pcrarraydataanalysis.php). The average expression of the most stable housekeeping gene (*ACTB*) in the array (the ‘normalization’ gene) was used to calculate ΔCt values. ΔΔCt values in the non-irradiated and irradiated groups were determined, and the fold change was calculated as 2^(−ΔΔCt)^.

### Statistical analysis

All values were from three to eight animals per datapoint and are reported as mean ± standard error of the mean. The Jonckheere–Terpstra trend test was performed to non-parametrically detect monotone trends in the proportion of Ki67-positive cells relative to age or time after irradiation. One-way analysis of variance (ANOVA) with subsequent *post hoc* Dunnett's multiple comparison testing was performed to compare the proportion of Ki67-positive cells, number of 53BP1 foci, LC3-II/LC3-I ratio, and expression of p62 in different age groups and at different times after irradiation. All *P*-values are two-sided; those less than 0.05 are considered statistically significant. All statistical analyses were performed using SAS statistical software, version 9.2 (SAS Institute, Cary NC).

## RESULTS

### Expression of Ki67 in thyroid follicular epithelial cells after irradiation of rats of different ages

**Fig. 1.**
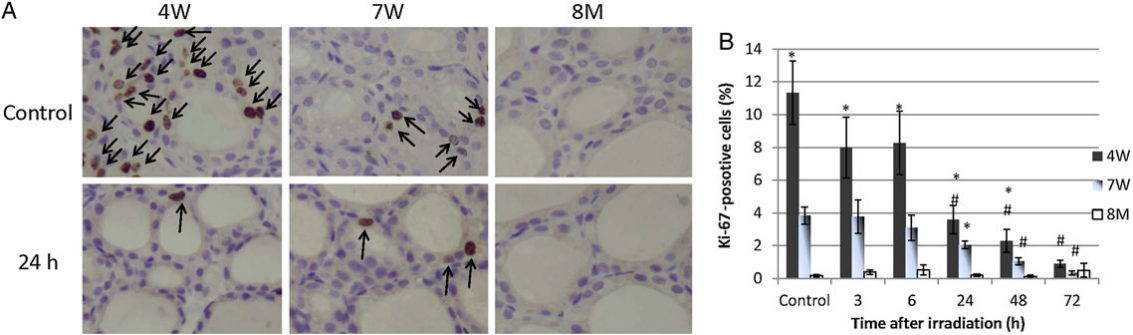
Ki67 immunohistochemistry in non-irradiated (control) and irradiated 4W, 7W and 8M thyroid follicular epithelial cells ( × 400 magnification) **(A**), and proportion of Ki67-positive cells in thyroid follicular epithelial tissue after irradiation (**B**). Ki67-positive cells are indicated by the arrows. Data are presented as the mean ± standard error of the mean of the results for three to eight rats per datapoint. # indicates *P* < 0.05 compared with thyroid tissue of non-irradiated rats. * indicates *P* < 0.05 compared with thyroid tissue of 8M rats.

To evaluate proliferation after irradiation, immunohistochemical staining for the proliferative marker Ki67 was performed. Representative images of Ki67 expression are presented in Fig. 1A. The percentages of Ki67-positive cells in non-irradiated thyroid follicular tissues of 4W, 7W and 8M rats were 11%, 3.8% and 0.2%, respectively (Fig. 1B). Non-parametric analysis using the Jonckheere–Terpstra test revealed a tendency toward an increase in the proportion of Ki67-positive cells in non-irradiated rats with decreasing age (*P* < 0.0001); the proportion of proliferating cells was significantly higher in the 4W group than the 8M group (*P* < 0.05). Non-parametric analysis also showed a tendency toward a decrease in the proportion of Ki67-positive cells after irradiation in the 4W and 7W groups (*P* < 0.0001). The proportion of Ki67-positive cells was significantly decreased 24, 48 and 72 h after irradiation in the 4W group and at 48 and 72 h in the 7W group compared with non-irradiated cells (0 h). In contrast, the proportion of Ki67-positive thyroid follicular cells in the 8M group remained unaltered up to 72 h after irradiation.

### Number of 53BP1 foci in thyroid follicular epithelial cells after irradiation of rats of different ages

**Fig. 2.**
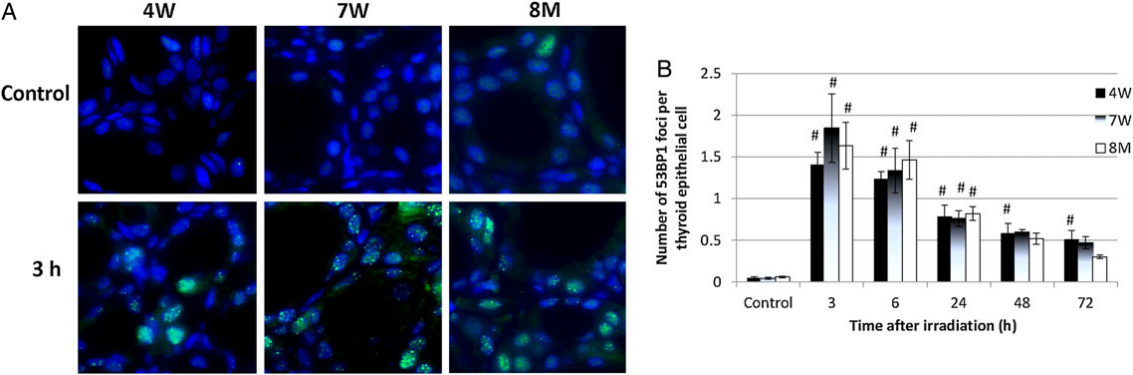
Immunofluorescent staining of p53 binding protein 1 (53BP1) in non-irradiated (control) and irradiated 4W, 7W and 8M thyroid follicular epithelial cells ( × 1000 magnification) **(A)**, and the average number of 53BP1 foci per thyroid epithelial cell after irradiation **(B)**. Data are presented as the mean ± standard error of the mean of the results for three to seven rats per datapoint. # indicates *P* < 0.05 compared with cells of non-irradiated rats.

53BP1 foci were used as a marker of DNA damage. As shown in Fig. 2A, 53BP1 foci were observed in nuclei 3 h after IR. The average numbers of 53BP1 foci in the thyroid follicular cells of non-irradiated rats were similar in all age groups (Fig. 2B). The number of 53BP1 foci in the thyroid follicular cells of 4W, 7W and 8M rats peaked at 3 h after irradiation; it then decreased gradually until 72 h but remained statistically higher than the number of foci in non-irradiated cells of 4W rats at 48 and 72 h (Fig. 2B).

### Abundance of p53, phospho-p53^Ser15^, p21 and cleaved caspase 3 after irradiation

**Fig. 3.**
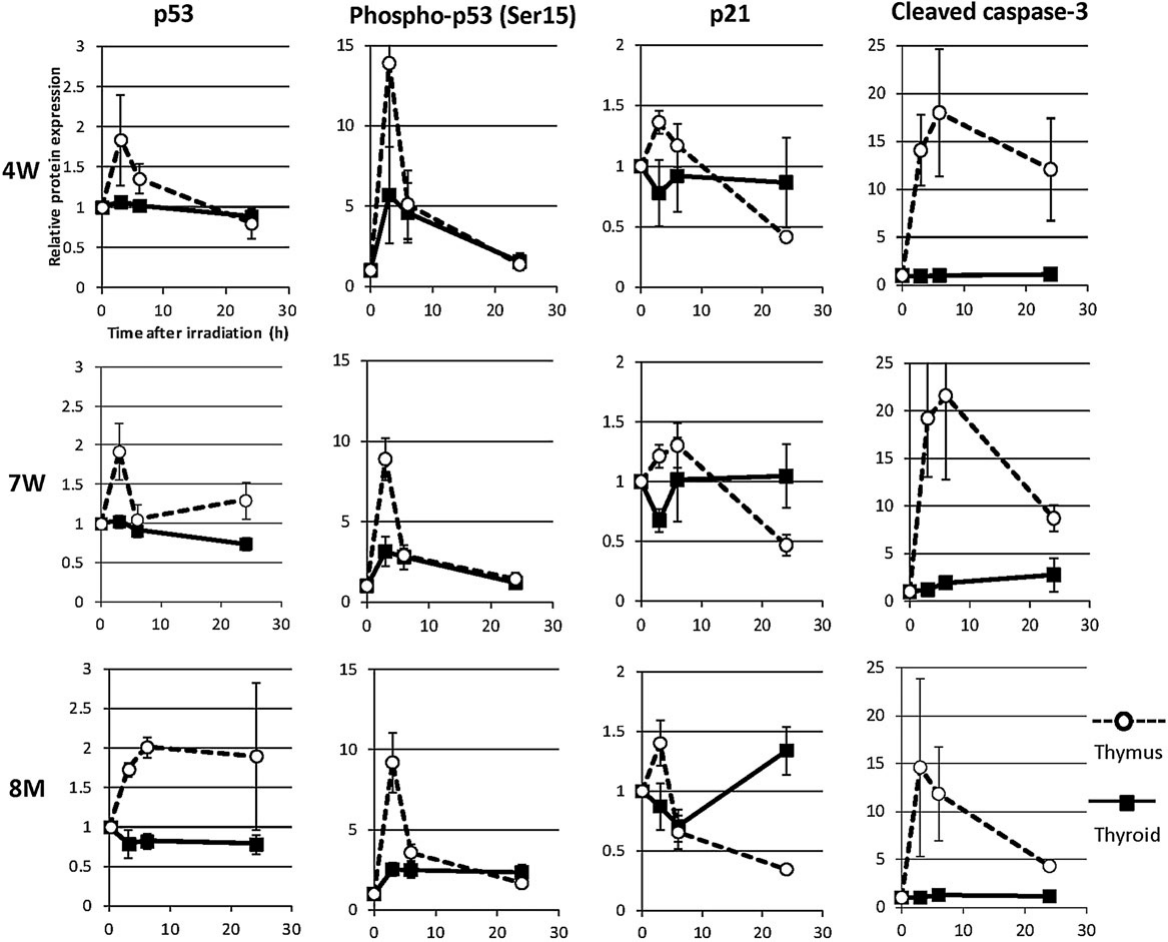
Amounts of p53, phospho-p53^ser15^, p21 and cleaved caspase 3 in the thyroid and thymus glands of non-irradiated (0 h) and irradiated (3 to 24 h) 4W, 7W and 8M rats were determined via western blotting. Protein expression was quantified via densitometric analysis and normalized to actin. Data are expressed as the ratio of the irradiated value to the non-irradiated value. Solid lines indicate thyroid. Dotted lines indicate thymus. Data are presented as the mean ± standard error of the mean of the results for three to five rats per datapoint.

Western blots were performed to determine the effect of IR on the abundance of DNA damage response proteins in the thyroid and thymus (positive control); amounts of protein were quantified via densitometry. Kinetic diagrams are shown in Fig. 3. In the thymus, levels of p53, phospho-p53^Ser15^, p21 and cleaved caspase 3 were increased at 3 and 6 h in the 4W, 7W and 8M groups. In contrast, in the thyroid, levels of phospho-p53^Ser15^ were increased slightly at 3 and 6 h, whereas levels of p53, p21 and cleaved caspase 3 did not change regardless of age up to 24 h after irradiation.

### Radiation-induced apoptosis of thyroid follicular epithelial cells after irradiation of rats of different ages

**Fig. 4.**
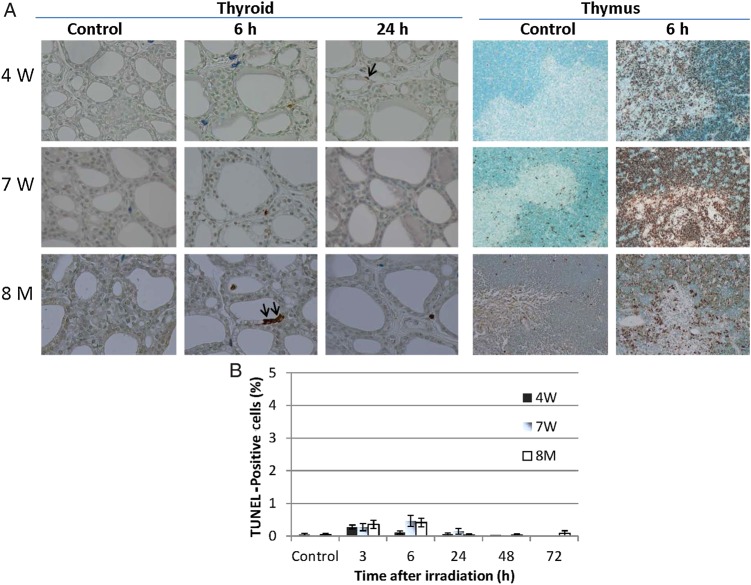
Terminal deoxynucleotidyl transferase-mediated dUTP nick end labeling (TUNEL) staining of non-irradiated (control) and irradiated 4W, 7W and 8M thyroid glands ( × 400 magnification) and thymus glands ( × 200 magnification) (**A**), and proportion of TUNEL-positive cells in thyroid follicular epithelial cells after irradiation (**B**). TUNEL-positive cells in thyroid follicular epithelial cells are indicated by the arrows. Data are presented as the mean ± standard error of the mean of the results for three to seven rats per datapoint.

To detect radiation-induced apoptosis of thyroid follicular cells and thymic lymphocytes (positive control), TUNEL staining was performed. A large number of apoptotic cells was observed in the thymic lymphocytes of 4W, 7W and 8M rats (Fig. 4A). In contrast, only a few apoptotic cells were seen in the thyroid follicular cells of irradiated rats 3–72 h after irradiation (Fig. 4A and B). Although a few TUNEL positive cells were also observed in the mesenchymal cells and in the colloids of irradiated thyroids, these were not counted.

### LC3-II/LC3-I ratio and p62 expression

**Fig. 5.**
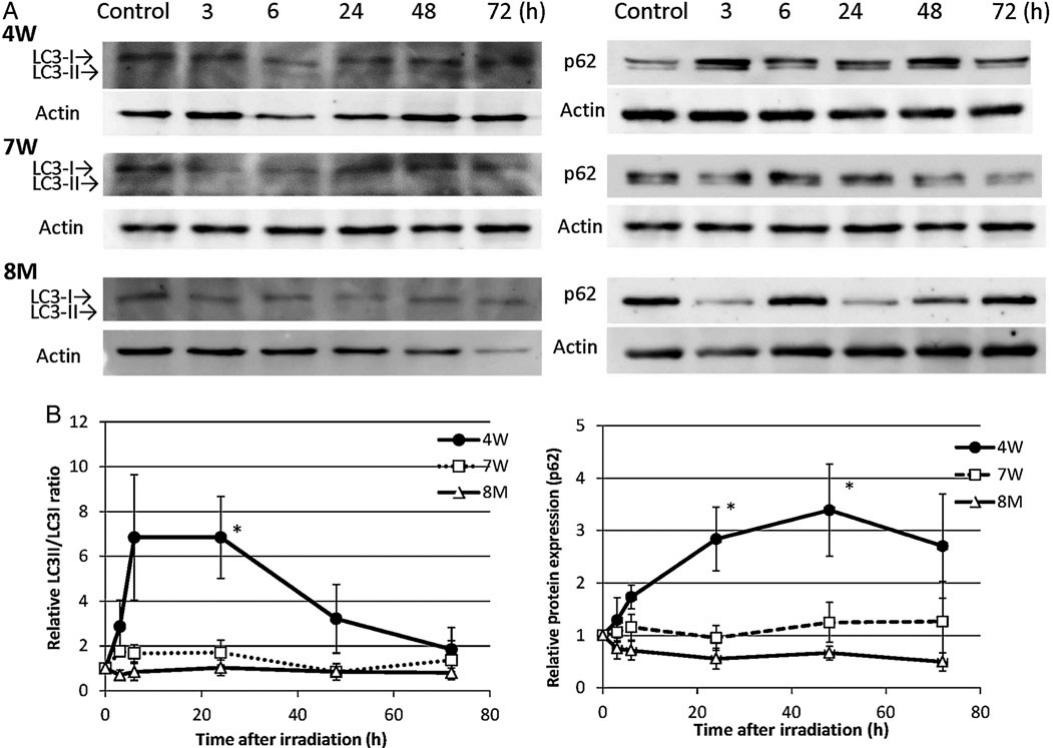
Western blots for LC3 and p62 in non-irradiated (control) and irradiated (3 to 72 h) 4W, 7W and 8M thyroid glands **(A)**, and ratios of LC3-II to LC3-I and expression of p62 (**B)**. LC3-II/LC3-I ratios in irradiated thyroids were normalized to LC-II/LC3-I ratios in non-irradiated controls. p62 levels were normalized to actin levels and are expressed as the ratio of the irradiated value to the non-irradiated value. Data are presented as the mean ± standard error of the mean of the results for three to seven rats per datapoint. * indicates *P* < 0.05 compared with 8M thyroids.

To determine whether autophagy was induced in 4W, 7W and 8M thyroid glands after irradiation, levels of LC3-II and p62, which are markers for autophagy, were examined via western blotting. Protein expression is shown in Fig. 5A, and kinetic diagrams are shown in Fig. 5B. The LC3-II/LC3-I ratio increased progressively after irradiation of 4W thyroids and was 6.8-fold higher than baseline at 24 h. It did not increase after irradiation of 8M thyroids. Consequently, there was a significant difference in LC3-II abundance between 4W and 8M thyroids at 24 h (*P* < 0.05). The LC3-II/LC3-I ratio in 7W thyroids increased slightly during the 24 h after irradiation, but there was no significant difference between the 7W and 8M groups. p62 expression increased progressively after irradiation of 4W thyroids and was 3.4-fold higher than baseline at 48 h. It did not increase in irradiated 7W or 8M thyroids. There was a significant difference in p62 expression between 4W and 8M thyroids at 24 h and 48 h after irradiation (*P* < 0.05).

### Expression of genes related to autophagy after irradiation

The expression profiles of autophagy-associated genes in the thyroids of 4W and 8M rats 24 h after irradiation were compared with those of non-irradiated age-matched controls (Table 1). The expression of several autophagy-related genes increased (e.g. *Atg16l2*, *Atg9a*, *Ctss*, *Irgm and Wipi1*) or slightly increased (*Atg12*, *Atg16l1*, *Atg5* and *Maplc3a*) in 4W thyroids after irradiation. On the other hand, only two autophagy-related genes, *Ctss* and *Wipi 1*, increased to a lesser extent in 8M irradiated thyroids. The expression of several genes involved in the co-regulation of autophagy and apoptosis (e.g. *Dapk1, Fas* and *Casp3*) and mitochondrial function (e.g. *Pink1*) was greater in 4W and 8M irradiated thyroids than in age-matched non-irradiated thyroids. The expression of several genes involved in the co-regulation of autophagy and apoptosis (e.g. *Bax* and *Tnfsf10*)*,* and the co-regulation of autophagy and cell cycle (e.g. *Mapt*) increased in 4W irradiated thyroids.

**Table 1.** The expression of genes associated with autophagy in thyroids obtained from 4W and 8M rats 24 h after irradiation was analyzed using pathway-specific PCR array. Fold change represents the average of triplicate experiments from single rats. The bold numbers indicate a more than 2-fold increase. *P < 0.05 compared with non-irradiated group, unpaired Student *t* test.

Gene symbol	Gene name	Fold change	
		4W rat	8M rat
*Atg12*	Autophagy-related 12 homolog (*S. cerevisiae*)	1.5	0.57*
*Atg16l1*	Autophagy-related 16-like 1 (*S. cerevisiae*)	1.58	0.95
*Atg16l2*	Autophagy-related 16 like 2 (*S.cerevisiae*)	3.32*	0.96
*Arg5*	Autophagy related 5 homolog (*S. cerevisiae*)	1.43	0.87
*Atg9a*	Autophagy-related 9A (yeast)	**2.08**	1.39
*Bax*	Bcl-associated X protein	3.09*	1.38
*Bcl-2*	B-cell CLL/lympnoma 2	1.2	0.72*
*Casp3*	Caspase 3	1.69	1.67*
*Ctss*	Cathepsin S	3.25*	1.94*
*Dapk1*	Death associated protein kinase 1	**2.32**	**2.09**
*Fas*	Fas (TNF receptor superfamily, member 6)	6.06*	6.35*
*Irgm*	Immunity-related GTPase family M	2.46*	0.92
*Map1lc3a*	Microtubule-associated protein 1 light chain 3 alpha	1.82	1.04
*Mapk14*	Mitogen-activated protein kinase 14	1.16	0.72*
*Mapt*	Microtubule-associated protein tau	1.86*	0.56*
*Pink1*	PTEN induced putative kinase 1	**2.06**	2.52*
*Sqstm1*	Sequestosome 1	1.19	0.77
*Tnfsf10*	Tumor necrosis factor (ligand) superfamily, member10	1.99*	1.23
*Wipi1*	WD repeat domain, phosphoinositide interacting 1	1.95	1.61*

## DISCUSSION

The acute radiation response of thyroid follicular epithelial cells in rats of different ages was investigated. In non-irradiated rats, the 4W group had the highest proportion of Ki67-positive thyroid follicular cells compared with the other age groups. The number of proliferating cells in 4W and 7W thyroid follicular tissues was significantly decreased 72 h after irradiation. In contrast, there was no decrease in the percentage of proliferating cells in 8M thyroids after irradiation (Fig. 1).

The DNA damage response that occurs following IR (e.g. cell cycle arrest and DNA repair, senescence, or apoptosis) is primarily controlled by the p53 tumor suppressor protein [9]. In rat thymus glands, in which numerous apoptotic cells were observed, levels of p53, phospho-p53^Ser15^, p21 and cleaved caspase 3 increased after irradiation regardless of age. However, in thyroid glands, the level of phospho-p53^Ser15^ only slightly increased after irradiation (at 3 h), while no increase was detected in the levels of p53, p21 or cleaved caspase 3 to 24 h after irradiation regardless of age (Fig. 3). Apoptosis was not induced after irradiation of thyroid follicular cells in any age group. This was in contrast to the thymus, a highly radiosensitive organ, in which numerous apoptotic cells were seen (Fig. 4). This result indicates that unlike the thymus, neither p53 accumulation nor apoptosis was induced in the thyroid gland after irradiation. A recent study suggested that phospho-p53^Ser15^ interacts with 53BP1 and ATM phosphorylated at serine 1981 and localizes to sites of IR-induced DNA damage [26]. Zhang *et al.* suggested two-phase dynamics for p53 in the DNA damage response and that p53-mediated cell cycle arrest primarily involved p53 phosphorylated at serine 15 and serine 20 [9]. In our study, the number of 53BP1 foci and the level of phospho-p53^Ser15^ in the thyroid gland were increased irrespective of age at 3 h after irradiation (Figs 2 and 3). These results suggest that the magnitude of the DNA damage was similar in immature, young adult and adult rat thyroid glands after irradiation. However, the number of 53BP1 foci in the 4W group remained high at 48 h and 72 h after irradiation (Fig. 2). This result suggests that repair is slightly delayed in 4W thyroid glands, which may contribute to radiation-induced thyroid carcinogenesis.

Amounts of LC3-II and p62, which are markers of autophagy, increased in 4W thyroid glands from 6 to 72 h after irradiation but did not increase in 8M thyroids (Fig. 5). The expression of the autophagy-related genes *Atg16l2*, *Atg9a*, *Ctss*, *Irgm, Mapt, Wipi1* and *Bax* increased in 4W thyroid glands after irradiation. In contrast, the expression of apoptosis-related genes, including *Fas*, *Dapk1* and *Casp3,* increased in both 4W and 8M thyroid glands after irradiation (Table 1).

The formation of the Atg12-5-16L1 complex is essential for autophagosome formation. Atg16L2 is a novel isoform of Atg16L with the same domain structure as Atg16L1. It forms a complex with Atg12-conjugated Atg5 and has E3-like activity in regard to LC3 lipidation [27]. ATG9 is thought to be involved in autophagy initiation, as well as in the delivery of lipids to growing phagophores [28]. In this study, we demonstrated that the expression of proteins and genes associated with autophagy increased in immature, but not adult, thyroid glands after irradiation. However, no apoptosis was detected, despite the expression of pro-apoptotic genes in 4W and 8M thyroid glands.

Autophagy serves to direct damaged proteins and organelles to lysosomes for degradation. It functions as a homeostatic mechanism that influences both protein and genomic integrity. Ryan suggested that p53 activates a spectrum of target genes that positively regulate autophagy [29]. Some p53 target genes involved in autophagy regulation, such as *Dram1*, *ISG20L1*, *DAPK-1*, *Bax* and *PUMA*, have also been implicated in cell death [29]. Our data suggest that apoptosis is inhibited, while autophagy is promoted, following irradiation of the immature thyroid.

It has been suggested that excess or prolonged autophagy can promote an alternative mechanism leading to programmed cell death, which is particularly relevant in cancers defective in apoptosis [30]. Furthermore, RAD001, a potent activator of autophagy, enhances the therapeutic response to cytotoxic chemotherapy and external beam radiation in papillary thyroid cancer [31]. It has also been suggested that autophagy is potentially tumor suppressive at the initial stages of cancer development [32].

The hypersensitivity of thyroid glands in children to radiation-induced cancers may be due to p53 dysfunction and defective apoptosis, despite high proliferative activity in immature thyroid follicular epithelial cells after exposure to IR. The relationship between acute radiation-induced autophagy and thyroid cancer in immature thyroids is unclear. Our results suggest that autophagy is induced in immature rat thyroid follicular cells up to 72 h after irradiation. Thus, autophagy may be a marker of the DNA damage response to irradiation in the immature thyroid. Further study is necessary to determine the role of autophagy in irradiated thyroid follicular epithelial cells beyond 72 h. A future study examining whether thyroid epithelial cell damage becomes more severe and/or thyroid cancer is promoted if radiation-induced autophagy is inhibited may contribute to the elucidation of radiation-induced thyroid carcinogenesis.

The present study showed that the number of proliferating cells in immature and young adult thyroids, which exceeded the number of proliferating cells in adult thyroids, significantly decreased after irradiation. Amounts of DNA damage response molecules increased irrespective of age in irradiated thyroids. No apoptosis or accumulation of p53 was noted, despite the expression of pro-apoptotic genes in all age groups. Because IR induced autophagy in immature, but not adult, rat thyroids, we suggest that autophagy may be a marker of the DNA damage response to irradiation in immature thyroids.

## FUNDING

This work was supported in part by Grants-in-Aid for Young Scientists (B) (No. 24710062) from the Ministry of Education, Culture, Sports, Science, and Technology, Japan. Funding to pay the Open Access publication charges for this article was provided by Grant of Atomic Bomb Disease Institute at Nagasaki University, Nagasaki, Japan.
